# A coccidiostat can be replaced by or combined with a blend of direct fed microbials and xylanase in broiler production

**DOI:** 10.1016/j.psj.2025.105655

**Published:** 2025-08-06

**Authors:** Basheer Nusairat, Rasha Qudsieh, Jeng-Jie Wang

**Affiliations:** a Department of Animal Production, College of Agriculture, Jordan University of Science and Technology, Ar Ramtha 3030 Irbid, Jordan; b Novus International, Inc., Chesterfield MO 63005, USA

**Keywords:** Probiotic, Coccidiostat, Broiler, Lesion score, Gut health

## Abstract

The poultry industry continues to seek effective alternatives to coccidiostats that promote health and performance without compromising economic viability or result in antibiotic resistance. This study was designed to investigate the effect of a multi-strain Bacillus spp. probiotic in the presence of xylanase (DFM), and a monensin coccidiostat in a 2 × 2 factorial dietary treatment arrangement testing 2 levels of DFM (0 and 100 g/MT), and 2 levels of monensin (0 and 90 g/MT) on growth performance, gut lesions, environmental Clostridium perfringens load, and digesta pathogens load of broiler chickens (E. tenella, Salmonella spp., total aerobic count cells (APC), E. coli, and C. perfringens) with 10 replicate pens per treatment and 52 birds per replicate. Data were analyzed as two-way ANOVA. Results indicated that both DFM and monensin significantly improved body weight gain (P = 0.01, P = 0.0002, respectively), while monensin improved feed conversion ratio by 3 points (P = 0.01) by day 42. Lesion scores were lower in birds supplemented with the combination of DFM and monensin at both 21 d (P = 0.003) and 42 d (P = 0.007) compared to non-supplemented birds. Monensin reduced (P = 0.01) litter moisture by approximately 1% compared to non-supplemented diets. Pathogens load in digesta were suppressed further by the combination of DFM and monensin at 21 d for APC (P = 0.002), and 42 d for E. coli (P = 0.01), C. perfringens (P = 0.0003), E. tenella (P = 0.05), and Salmonella spp. incidence (P = 0.03). These findings suggest that DFM can be used as an alternative to monensin, or as part of a bio-shuttle program with monensin, for improving broiler performance, gut health, and environmental microbial safety.

## Introduction

Diverse food-animal production systems have been continuously seeking alternatives to antibiotic growth promoters (Huyghebaert et al., 2011; Rahman et al., 2022; Azizi et al., 2024). The ban on non-therapeutic use of antibiotics in animal feed continues since its initiation by the European Union (Castanon, 2007), leading to an increased use of coccidiostats (Granstad et al., 2020) which are still allowed in most countries. Monensin is the first carboxylic polyether ionophore introduced in the USA for the control of coccidiosis, caused by the Eimeria protozoa, in 1971 (Chapman et al., 2010) but it may also have antiviral and antibacterial properties (Kevin et al., 2009) such as against *Clostridium perfringens*, which causes necrotic enteritis. Furthermore, when used at subtherapeutic levels, ionophores have been shown to increase weight gain in chicken (Roila et al., 2019). However, coccidiosis economic burden is still estimated between $7 and $13 billion annually (Blake et al., 2020), and it is a very challenging disease to control, it is also considered one of the predisposing factors for necrotic enteritis which exacerbates the disease burden. Eimeria strains may develop resistance to some drugs which necessitates the use of multiple coccidiostats in rotation, furthermore, drug residues in tissues presents a public health concern (Martins et al., 2022). Therefore, there has been a growing interest in non-antibiotic feed additives, including alternatives to coccidiostats, that improve the performance of the birds and the gut, without developing resistance. Probiotics are among the promising alternatives to antibiotics which target health of the host rather than attacking the pathogen directly. Probiotics may work by colonizing the intestine thus positively influencing the intestinal microbiota composition, improving the immune function, producing certain metabolites, or competitively excluding other microbes (Kabir, 2009). Probiotics require nutrients in order to proliferate and exert their effect in the gut, these can be acquired from the feed with the aid of exogenous carbohydrases such as xylanases, leading to better utilization of feed ingredients, and at the same time providing the required nutrients to the probiotics, i.e. prebiotics. The enhancement in bird performance with xylanase addition can be attributed to the viscosity reduction, removal of cage effect by the degradation of cell wall non-starch polysaccharides (NSP), releasing entrapped nutrients, and a prebiotic effect of the xilooligosaccharides (XOS) degradation products generated. The unique combination of direct fed microbials (DFM) with enzymes, and the prebiotics produced by the action of such products, can lead to leveraging survival and activity of beneficial gut microbiota.

In addition to the awareness about diverting from antibiotics in farming practices, producers are also embracing sustainable practices to ultimately create a healthy environment that is an integral component of the one health concept to ultimately reduce antibiotic resistance in the food chain (WHO, 2024). For example, in 2015 the Norwegian broiler industry started phasing out coccidiostats from feed due to the concerns over their association with resistance against antimicrobials used in human medicine (Nilsson et al., 2012; VKM, 2015; Simm et al., 2019). Flock health is one reflection of environmental health; therefore, it is important to identify potential non-antibiotic alternatives that can support sustainable poultry production, and at the same time, enhance flock performance and control coccidiosis. Several studies have investigated probiotics as an alternative to ionophores; however, literature is scarce when it comes to investigating the combination effect between probiotics and ionophores (Trela et al., 2020) with no literature on multi-strain probiotic combination with ionophores, which could help in designing bio-shuttle programs aimed at controlling coccidiosis, and subsequently, necrotic enteritis. It is hypothesized that the supplementation of a combination of probiotics and xylanase could introduce a sustainable alternative to coccidiostats for the control of coccidiosis in poultry. Therefore, the purpose of this trial was to investigate the effect of supplementing a probiotic feed solution with and without monensin coccidiostat on growth performance, gut lesions, environmental *Clostridium perfringens* load, and pathogens load in the digesta of broiler chickens raised on used litter and fed corn-SBM based diets to 42 days of age.

## Materials and methods

This trial was conducted following the principles and guidelines in Guide for the Care and Use of Agricultural Animals in Research and Teaching (FASS, 2020) to ensure comfort of the birds.

### Experimental design

A total of 2,080 Ross 708 mixed sex 1-d old broilers were obtained from a commercial hatchery and randomly placed in floor pens (3 cm x 1.4 m) with 10 replicate pens per treatment, each containing 52 chicks and raised to 42 days of age, under commercial conditions, in a completely randomized design with 2 × 2 treatment factorial arrangement. Chicks were reared on a used litter spiked with 10^7^ CFU/bird of *C. perfringens* at d 1, and *Eimeria* spp at d 7 (*E. acervulina*: 10^4^ oocyst/bird, and *E. tenella*: 10^6^ oocyst/bird), and at d 10 for *E. maxima* (10^3^ oocyst/bird). Birds were given *ad libitum* access to feed and water. The lighting program included 23 hours of light for the first week, followed by 21 hours of light until 21 days, and 18 hours of light until 42 days.

### Experimental diets

A total of four, factorially arranged, dietary treatments were utilized to evaluate the effect of DFM and monensin when fed alone or as a combination. A 2 × 2 treatment factorial arrangement was used to investigate 2 levels of DFM (0 and 100 g/MT) and 2 levels of monensin (0 and 90 g/MT). The novel combination used in this trial contained a high-activity monocomponent xylanase product and 2 different *Bacillus* spp (EnzaPro^TM^ Feed Solution, Novus International, Inc., St. Louis, MO, USA) that provided 1 × 10^5^ CFU and 10 XU of *Bacillus* spp and endo-1,4-beta-xylanase, respectively per g of feed. Coban 90, a type A medicated article (Elanco Animal Health, Greenfield, IN, USA) was used as the coccidiostat and provided 90 g of monensin per ton. Both products were supplemented continuously throughout the study. An *in vitro* test was performed to ensure DFM and monensin were compatible prior to running the trial (data not shown), and results showed that the *Bacillus* spp. enumeration was not reduced when incubated with monensin at the manufacturer recommended dose for broilers. Table 1 illustrates the dietary formulation and nutrient composition for the corn-SBM basal starter, grower, and finisher phases. Diets were formulated to either meet or exceed nutrient requirements of broilers, and according to strain producer nutrition specification recommendation (Aviagen, 2022a). Diets did not contain any additional exogenous enzymes or antibiotic growth promoters (AGPs). Birds were fed a mash starter (d 1 to 21), grower (d 22 to 35), and finisher diets (d 36 to 42). Both xylanase activity and *Bacillus* spp. enumeration were confirmed by analyzing the feed samples for activity. Diets were also analyzed following AOAC (2006), for dry matter (934.01), crude protein (984.13, Kjeldahl), crude fat (920.39, by ether extraction), crude fiber (978.10), and ash (942.05).

**Table 1:** Composition and nutrient content of experimental diets.

Ingredient (%)	Starter	Grower	Finisher
	(1 – 21 d)	(22 – 35 d)	(36 – 42 d)
Corn	58.15	64.49	68.93
Soybean meal 48 %	36.30	29.49	23.34
Poultry meal	0.71	1.73	3.83
Poultry fat	0.05	0.05	0.05
DL- Methionine	0.25	0.19	0.08
Sodium chloride	0.49	0.45	0.39
Lysine	0.00	0.04	0.03
Limestone	1.60	1.32	1.22
Dicalcium phosphate	1.89	1.68	1.57
Vitamin and mineral premix^1^	0.50	0.50	0.50
Sand filler^2^	0.06	0.06	0.06
Calculated nutrients (%)			
Metabolizable energy (kcal/kg)	2900	3000	3100
Crude protein	22.0	20.0	19.0
Crude fat	1.44	1.87	1.81
Crude fiber	2.62	2.59	2.55
Ash	7.84	7.46	8.77
Calcium	1.05	0.9	0.85
Available phosphate	0.50	0.45	0.42
Sodium	0.22	0.2	0.18
SID^3^ lysine	1.28	1.15	1.02
SID methionine	0.54	0.50	0.44
SID methionine + cysteine	0.95	0.85	0.76
SID threonine	0.82	0.75	0.65
SID tryptophan	0.21	0.18	0.16
SID arginine	1.35	1.25	1.10
SID isoleucine	0.84	0.77	0.70
SID valine	0.96	0.89	0.80
Analyzed nutrients (%)			
Crude protein	22.8	18.9	17.8
Crude fat	1.70	1.57	1.67
Crude fiber	3.01	2.87	2.72
Ash	6.55	6.10	7.95

1 Vitamin and trace mineral premix supplied the following per kg of diet: 5,512 IU vitamin A, 1,852 IU vitamin D3, 11 IU vitamin E, 0.06 mg vitamin B12, 0.23 mg biotin, 1.87 mg menadione (K3), 0.44 mg thiamine, 3.75 mg riboflavin, 5.95 mg d-pantothenic acid, 1.32 mg vitamin B6, 34.17 mg niacin and 0.22 mg folic acid, for mineral supplied the following per kg of diet: Manganese: 120 mg, zinc: 120 mg, Iron: 80 mg, Copper: 10 mg, Iodine, 2.5 mg, Cobalt, 1 mg.

2 Sand filler used to allow for EnzaPro incorporation in 100 g/MT DFM treatments by replacing 0.01 % (100 g/MT) and/or Coban 90, as a source of monensin (0.05 %) of sand at each phase.

3 SID: standardized ileal digestible.

### Data collection

#### Growth performance

Birds and feed were weighed at placement and at 21, 35, and 42 days for growth performance parameters determination. Mortality was collected and recorded as it occurred. Measurements were used for determining body weight (BW), body weight gain (BWG), feed consumption (FC), feed conversion ratio (FCR) adjusted for mortality, and percent mortality.

#### Apparent metabolizable energy determination

The AME was determined based on fecal sample collections at the ages of 21 and 42 d. Birds were selected based on phenotypic appearance to differentiate males and females and randomly choose 2 males and 2 females per pen to place in raised-wire cages. At each collection period, birds were fasted for 6 hours prior to feeding their respective diets. Feed consumption was measured per cage, and all excreta were collected, dried, and ground. Both dried feed and excreta were analyzed for dry matter (AOAC, 2006; 934.01), gross energy (Parr oxygen bomb calorimeter, 6200 Isoperibol Calorimeter, Parr Instrument Company, Moline, IL), and nitrogen (AOAC, 2006; 984.13 (A-D)). The following calculations were used to determine apparent metabolizable energy (AME) and nitrogen-corrected (AMEn) retention (Hill and Anderson, 1958):

AME(kcal/kg)=[FCxGEfeed)−(DMfecalxGEfecal)]/FC

Nitrogenretention(NR)=[(FCxNfeed)−(DMfecalxNfecal)]

AMEn(kcal/kg)=AME−(NRx8.22)

Where: FC = feed consumption; GEfeed = gross energy of feed; DMfecal = fecal dry matter; GEfecal = gross energy of feces; NR = nitrogen retention

#### Microbial enumeration and incidence of the gastrointestinal tract contents

Microbial measurements were performed on 21 and 42 d. A total of 4 birds (2 males and 2 females) were sampled per pen, sex of birds was confirmed after sampling. *E. tenella* were enumerated using the McMaster technique in cecal contents and results were expressed as log_10_ oocyst per bird. The intestinal digesta contents were used for enumeration of *E. coli*, aerobic plate count (APC) using 3 M Petrifilm E. coli/Coliform count plates (incubated under aerobic conditions at 35°C for 24 hours), and *C. perfringens* (expressed as log_10_ CFU/g) following the procedure of Bacteriological Analytical Manual (Rhodehamel and Harmon, 1998), as well as determining the incidence of *Salmonella* spp. (expressed as %) following the procedure of Bacteriological Analytical Manual (Andrews and Hammack, 2007).

#### Intestinal lesion score

At 21 and 42 d, 4 birds from each pen (2 males and 2 females) were randomly selected, while taking into consideration the visual differences between males and females, for the purpose of intestinal lesion score evaluation. Birds were humanely sacrificed by neck dislocation, their sex was confirmed, and entire intestines (small and large intestines) were excised and scored by trained personnel. Lesions were evaluated as an indicator of coccidiosis caused by *Eimeria* spp. The scoring system illustrated by Johnson and Reid (1970) was followed based on the presence and/or severity of any intestinal lesions with the scoring range from 0 (no lesions found) to 4 (actual bleeding observed).

#### Litter moisture and C. perfringens load

Litter samples were collected per pen on days 0, 21 and 42 of age. Samples were taken from 3 sites of each pen and pooled together, and oven-dried following procedure in the AOAC (2006; 934.01) for litter moisture calculation, results were expressed as percentage (%).

For the same time points, counts of *C. perfringens* in litter were enumerated as an indicator of environmental pathogen load. The *C. perfringens* colony forming units (CFUs) per gram of litter were measured following the procedure of Bacteriological Analytical Manual (Rhodehamel and Harmon, 1998). Briefly, 25 grams of litter was homogenized in 225 mL of peptone diluent (0.1 % peptone), then 10-fold dilutions of each sample were prepared up to 10^9^. One (1) mL of each dilution was placed on Tryptose-sulfite-cycloserine (TSC) agar (TSA) plates and incubated under anaerobic conditions at approximately 35°C for approximately 24 hours. Plates were then removed from the incubator and total viable *C. perfringens* colonies were counted using dilution plates with approximately 20 – 200 CFUs. Samples were analyzed in quadruplicate.

### Statistical methods

Data were analyzed as a two-way ANOVA with a 2 × 2 treatment factorial arrangement using a completely randomized design with 10 replicate pens per dietary treatment and 20 replicates per main effect (DFM and monensin). The General Linear Model of SAS (The SAS System, 2013) was employed. Means were separated by LSMEANS. An arc-sin transformation was applied to the percentage data before testing for differences. Superscripts were determined based on PDIFF values. The intestinal lesion score data was analyzed using the nonparametric Kruskal-Wallis test followed by Dunn’s test for pairwise comparisons in JMP Pro 17. The experimental unit was the pen. Means were considered significantly different at a set *P* value of ≤ 0.05 while trends were set at 0.05 < *P* < 0.10.

## Results

### Growth performance

The results of growth performance are presented in Table 2. There was no interaction effect between DFM and monensin on any of the measured growth performance parameters (FC, BWG, FCR, and mortality). Results are reported for the starter (1 – 21 d), grower (22 – 35 d), finisher (36 – 42 d), and overall (1 – 42 d). Feed consumption (FC) was not affected by the supplementation of DFM or monensin. However, the effects of DFM and monensin supplementations were significantly observed on BW (data not shown) and BWG. DFM tended (*P* = 0.08) to improve BWG from 1 – 21 d and improved (*P* = 0.01) BWG at 42 d by 1.4 %, while monensin supplementation improved BWG from 1 – 21 d (*P* = 0.01) and 22 – 35 d (*P* = 0.02), as well as a 2 % improvement in the overall 42 d period (*P* = 0.0002). The FCR was improved by the supplementation of either DFM (*P* = 0.02) or monensin (*P* = 0.004) from 1 – 21 d, while monensin supplementation continued with a tendency (*P* = 0.06) to improve FCR from 22 – 35 d and resulted in a 2.5 points improvement (*P* = 0.01) in 1 – 42 d FCR. Mortality was not affected by any of the dietary treatments. Overall BWG of the birds were in accordance with the Ross 708 broiler standard parameters (Aviagen, 2022b), however, the FCR was slightly higher than the strain standards due to feeding mash rather than pellets.

**Table 2:** Least-square means for feed consumption (FC), body weight gain (BWG), feed conversion ratio (FCR) corrected for mortality, and mortality for broilers raised to 42 d of age.

		FC, g/bird				BWG, g/bird				FCR, g:g				Mortality, %d			
		1-21	22-35	36-42	1-42	1-21	22-35	36-42	1-42	1-21	22-35	36-42	1-42	1-21	22-35	36-42	1-42
DFM (g/MT)	Monensin (g/MT)	Period (d)															
0		1044	2471	1369	4884	793^y^	1303	680	2777^b^	1.314^b^	1.799	1.860	1.713	1.3	0.3	0.1	1.7
100		1047	2467	1379	4910	802^x^	1320	693	2816^a^	1.302^a^	1.777	1.857	1.699	1.7	0.2	0.1	2.0
SE		5	17	27	22	4	8	12	10	0.003	0.010	0.037	0.006	0.3	0.1	0.1	0.4
	0	1042	2466	1383	4891	791^b^	1299^b^	679	2768^b^	1.314^b^	1.802^y^	1.873	1.719^b^	1.4	0.2	0.2	1.8
	90	1049	2471	1383	4903	804^a^	1325^a^	695	2825^a^	1.300^a^	1.775^x^	1.843	1.694^a^	1.6	0.3	0.0	1.9
	SE	5	17	27	22	4	8	12	10	0.003	0.010	0.037	0.006	0.3	0.1	0.1	0.4
0	0	1037	2472	1361	4870	784	1287	671	2742	1.321	1.821	1.864	1.728	1.1	0.3	0.2	1.6
0	90	1051	2469	1377	4897	802	1320	690	2812	1.306	1.779	1.855	1.699	1.5	0.4	0.0	1.9
100	0	1046	2460	1404	4911	797	1310	687	2794	1.309	1.783	1.881	1.710	1.7	0.2	0.2	2.1
100	90	1047	2474	1390	4910	807	1331	700	2838	1.294	1.771	1.831	1.689	1.7	0.2	0.0	1.9
SE		8	24	38	31	5	11	17	14	0.005	0.014	0.052	0.009	0.5	0.2	0.2	0.6
*P* value																	
DFM		0.73	0.86	0.46	0.39	0.08	0.13	0.45	0.01	0.02	0.11	0.95	0.12	0.41	0.28	0.99	0.71
Monensin		0.38	0.82	0.98	0.68	0.01	0.02	0.35	0.0002	0.004	0.06	0.57	0.01	0.70	0.90	0.17	0.99
DFM × Monensin		0.39	0.72	0.68	0.66	0.43	0.57	0.88	0.35	0.98	0.29	0.70	0.68	0.68	0.95	0.99	0.71

^a,b^Means in a column within each main effect that lack common superscript differ significantly (*P* ≤ 0.05).

^x,y^Means in a column within each main effect that lack common superscript tends to differ significantly (0.05 < *P* ≤ 0.10).

### Apparent metabolizable energy

The effect of DFM and monensin on AME and AMEn parameters are presented in Table 3. Both parameters were measured at 21 and 42 d. There was no interaction between DFM and monensin on energy retention. The basal diets investigated in this study did not contain any source of xylanase as illustrated in Table 1. Supplementing DFM improved (*P* < 0.0001) AME and AMEn at both 21 and 42 d by approximately 50 kcal/kg, which translates to approximately 1.6 % and 1.5 % increase in AME and AMEn, respectively, compared to non-supplemented treatment, regardless of age. On the other hand, supplementation of monensin did not have a significant effect on AME and AMEn at either age.

**Table 3:** Least-square means for apparent metabolizable energy (AME) and apparent metabolizable energy corrected for nitrogen (AMEn) for broilers raised to 42 d of age.

		AME		AMEn	
		D 21	D 42	D 21	D 42
DFM (g/MT)	Monensin (g/MT)	kcal/kg			
0		2903^b^	3100^b^	2871^b^	3071^b^
100		2948^a^	3146^a^	2918^a^	3116^a^
SE		4	4	4	4
	0	2923	3120	2892	3091
	90	2928	3125	2897	3095
	SE	4	4	4	4
0	0	2899	3097	2867	3068
0	90	2906	3103	2875	3074
100	0	2947	3144	2917	3115
100	90	2949	3147	2919	3117
SE		5	6	5	6
P value					
DFM		<0.0001	<0.0001	<0.0001	<0.0001
Monensin		0.40	0.46	0.38	0.55
DFM × Monensin		0.63	0.81	0.52	0.75

^a,b^Means in a column within each main effect that lack common superscript differ significantly (*P* ≤ 0.05).

### Gastrointestinal tract microbial load

Table 4 shows the data for *E. coli*, aerobic plate count (APC), *C. perfringens, E. tenella, Salmonella* spp, and intestinal lesion scores evaluated at both 21 and 42 d. Intestinal digesta contents were collected to determine *E. coli*, aerobic plate count (APC), and *C. perfringens* counts (log_10_ CFU/g) as well as *Salmonella* spp incidence (%). *E. tenella* was evaluated in cecal contents and reported as log_10_ oocyst/bird. The intestinal lesion scores were reported as an overall score of the intestines.

**Table 4:** Least-square means for intestinal digesta load of *E. coli*, aerobic plate count (APC), and *C. perfringens*, cecal load of *E. tenella*, Salmonella incidence in intestinal digesta, and overall intestinal lesion score measured at 21 and 42 d of age.

		*E. coli*		APC		*C. perfringens*		*E. tenella*		Salmonella incidence		Lesion score	
		D 21	D 42	D 21	D 42	D 21	D 42	D 21	D 42	D 21	D 42	D 21	D 42
DFM (g/MT)	Monensin (g/MT)	Log_10_ CFU/g						Log_10_ oocyst/bird		%		Score	
0		5.37^a^	5.39	7.62	7.60^a^	4.03^a^	4.09	2.33^a^	2.38	58.75^x^	52.50	0.63^a^	0.45
100		5.01^b^	4.82	7.09	7.13^b^	3.72^b^	3.60	2.12^b^	2.08	46.25^y^	36.25	0.34^b^	0.27
SE		0.11	0.11	0.09	0.13	0.08	0.06	0.06	0.06	4.62	4.32	0.07	0.06
	0	5.39^a^	5.48	7.75	7.64^a^	4.19^a^	4.10	2.39^a^	2.41	60.00^a^	52.50	0.64^a^	0.51^a^
	90	5.00^b^	4.73	6.96	7.09^b^	3.56^b^	3.59	2.06^b^	2.05	45.00^b^	36.25	0.32^b^	0.21^b^
	SE	0.11	0.11	0.09	0.13	0.08	0.06	0.06	0.06	4.62	4.32	0.07	0.07
0	0	5.66	5.99^a^	8.22^a^	8.00	4.44	4.50^a^	2.52	2.64^a^	70.00	67.50^a^	0.85^a^	0.67^a^
0	90	5.08	4.78^b^	7.01^b^	7.19	3.62	3.68^b^	2.14	2.11^b^	47.50	37.50^b^	0.40^ab^	0.22^b^
100	0	5.11	4.96^b^	7.28^b^	7.29	3.94	3.69^b^	2.26	2.17^b^	50.00	37.50^b^	0.43^ab^	0.34^ab^
100	90	4.91	4.68^b^	6.91^b^	6.98	3.50	3.51^b^	1.98	1.99^b^	42.50	35.00^b^	0.24^b^	0.20^b^
SE		0.15	0.15	0.13	0.18	0.12	0.08	0.08	0.09	6.54	6.11	0.10	0.09
*P* value													
DFM		0.02	0.001	0.0004	0.01	0.01	<0.0001	0.02	0.001	0.06	0.01	0.01	0.12
Monensin		0.02	<0.0001	<0.0001	0.004	<0.0001	<0.0001	0.0003	0.0002	0.03	0.01	0.007	0.004
DFM × Monensin^1^		0.22	0.01	0.004	0.17	0.10	0.0003	0.58	0.05	0.26	0.03	0.003	0.007

^a,b^Means in a column within each main effect that lack common superscript differ significantly (*P* ≤ 0.05).

1 For the DFMxMonensin interaction of the lesion score, the Kruskal-Wallis nonparametric analysis was conducted followed by Dunn’s pairwise comparison looking at the one-way treatments as a product of the 2 × 2 arrangement.

Supplementation of DFM reduced the load of *E. coli* at 21 d (*P* = 0.02), APC at 42 d (*P* = 0.01), *C. perfringens* at 21 d (*P* = 0.01), and *E. tenella* at 21 d (*P* = 0.02). It also tended (*P* = 0.06) to reduce the *Salmonella* spp incidence measured at 21 d. Furthermore, the intestinal lesion scores were lowered due to DFM supplementation at both 21 d (*P* = 0.01) and 42 d (*P* = 0.05) by approximately 46 % and 39 %, respectively compared to the non-supplemented control.

Supplementation of monensin reduced (*P* < 0.05) the counts of measured microorganisms at 21 d (*E. coli, C. perfringens*, and *E. tenella*) as well as APC 42 d count (*P* = 0.004), as well as the *Salmonella* spp incidence at 21 d (*P* = 0.03). Furthermore, intestinal lesion scores were reduced by monensin supplementation at both 21 d and 42 d by approximately 50 % and 58 %, respectively.

There was an interaction between DFM and monensin on the *E. coli* count at 42 d (*P* = 0.01), APC at 21 d (*P* = 0.004), *C. perfringens* at 42 d (*P* = 0.0003), *E. tenella* at 42 d (*P* = 0.05), and *Salmonella* spp incidence at 42 d (*P* = 0.03). There was also a tendency for interaction on *C. perfringens* at 21 d (*P* = 0.10). The interaction effect followed the same trend for all the variables where the non-supplemented control had significantly (*P* < 0.05) higher load (in the case of *E. coli*, APC, *C. perfringens*, and *E. tenella*) and incidence (in the case of *Salmonella* spp) compared to the treatments supplemented with either DFM, monensin, or their combination. Although it was not statistically separated, but the combination of DFM and monensin numerically reduced the counts and incidence further than each single component alone. *E. coli* counts at 42 d and APC at 21 d, were each reduced by 1.3 log_10_ CFU, while *C. perfringens* was reduced by 1 log CFU when both DFM and monensin were supplemented, compared to the non-supplemented control. *E. tenella* oocysts were reduced by 0.65 log at 42 d with the combined supplementation of DFM and monensin, while the *Salmonella* spp incidence at 42 d was reduced by 32.5 %, compared to the non-supplemented control.

The intestinal lesion scores measured at 21 and 42 d were significantly reduced by the combination of DFM and monensin. At 21 d, birds supplemented with the combination had a reduction in lesion score by 0.61 (*P* = 0.003) compared to the non-supplemented birds. Furthermore, at 42 d, the supplementation of monensin alone or in combination with DFM reduced (*P* = 0.007) intestinal lesion score compared to the non-supplemented birds.

### Litter moisture and *C. perfringens* load

The main effects for DFM and monensin supplementation on litter moisture (%) and *C. perfringens* counts (CFU/g) are presented in Table 5. Both parameters were measured at 0, 21 and 42 d. DFM supplementation did not affect (*P* > 0.05) litter moisture at any of the measured timepoints. However, monensin supplementation reduced (*P* = 0.01) litter moisture by 4 % compared to non-supplemented treatment at 42 d. For the counts of *C. perfringens* in the litter, DFM supplementation tended (*P* = 0.09) to reduce litter *C. perfringens* counts at 21 d with no effect at 42 d, while monensin supplementation reduced *C. perfringens* counts by 14 % and 12.5 % at 21 d (*P* = 0.01) and 42 d (*P* = 0.03), respectively, compared to non-supplemented control.

**Table 5:** Least-square means for litter moisture and *C. perfringens* load at 0, 21, and 42 d of age.

		Litter moisture			Litter *C. perfringens*		
		0	21	42	0	21	42
DFM (g/MT)	Monensin (g/MT)	%			Log_10_ CFU/g		
0		18.3	22.9	26.6	4.16	4.05^x^	3.85
100		18.4	22.6	26.0	4.29	3.69^y^	3.66
SE		0.19	0.20	0.27	0.18	0.14	0.13
	0	18.5	23.0	26.9a	4.36	4.17^a^	3.96^a^
	90	18.6	22.6	25.7^b^	4.08	3.56^b^	3.54^b^
	SE	0.19	0.20	0.27	0.18	0.14	0.13
0	0	18.6	23.0	27.4	4.29	4.47	4.08
0	90	18.7	22.9	25.8	4.03	3.62	3.61
100	0	18.4	22.9	26.4	4.44	3.86	3.85
100	90	18.5	22.3	25.6	4.14	3.51	3.48
SE		0.27	0.29	0.39	0.25	0.20	0.18
*P* value							
DFM		0.50	0.27	0.12	0.59	0.09	0.31
Monensin		0.74	0.25	0.01	0.27	0.01	0.03
DFM × Monensin		0.88	0.38	0.37	0.95	0.22	0.79

^a,b^Means in a column within each main effect that lack common superscript differ significantly (*P* ≤ 0.05).

## Discussion

This study investigated a probiotic potential as a standalone feed solution or in conjunction with monensin to enhance gut health in broilers, and to mitigate the pathogen load in both the host and the environment, leading to ameliorating the enteric disease burden for the poultry industry. The absence of interactions between the main effects on the majority of evaluated parameters indicated that these responses were due to either DFM or monensin supplementation and that these feed additives are independent from each other (additive effect) for the effects on growth performance, however, there were several significant interactions for the microbial load parameters. Overall, mortality was around 2 % per interaction treatment, which makes the mortality for the entire experiment slightly higher than what is commercially anticipated. This slight increase in mortality could be due to the mild microbial challenge employed through the litter. The measured BWG and FCR parameters were improved by supplementation of either DFM and monensin, while FC was comparable among treatments; similar results on FC were previously reported by Nusairat and Wang (2020; 2021) and Nusairat et al. (2022) for the effect of DFM in broilers. Moreover, Ghasemi-Sadabadi et al. (2020) reported that feed consumption was comparable between control and ionophore-supplemented birds from 1 to 42 d when comparing ionophore and non-ionophore coccidiostats in broilers. Having a comparable FC allows for interpreting the responses as being caused by the feed additive *per se* rather than the difference in the amount of intake of that specific additive. Body weight gain from 0 to 42 d was improved by supplementation of either DFM or monensin at a similar feed consumption, which supports that BWG was not confounded by variation in FC rather by supplementation of the feed additive. These findings are consistent with previous studies on direct fed-microbials and xylanase combinations (Harrow et al., 2007; Nusairat et al., 2018; 2022; Nusairat and Wang, 2020; 2021), and on ionophore-based coccidiostats supplementation (EFSA, 2019; Hooge et al., 2000) for body weight improvement. On the other hand, a study was conducted by Ribeiro et al. (2000) on the effect of monensin on broilers and showed that supplementing monensin did not affect body weight gain but improved FCR at 42 d. The improvements achieved by the probiotic supplementation could be related to beneficial modulation of the host microbiota, competitive exclusion of the opportunistic pathogens found in the gastrointestinal tract, or by directly influencing the immune system (Dalloul et al., 2003; Kulkarni et al., 2022).

The AME and AMEn measured at both 21 and 42 d were not affected by monensin, however DFM supplementation improved AME and AMEn. These results were expected since the basal diets did not contain any xylanase, and monensin does not work directly on feed ingredients to release energy, while the xylanase component in DFM was able to assist in releasing energy. It has been well-established that xylanases can hydrolyze the non-starch polysaccharides in plant cell walls (Adeola and Cowieson, 2011) leading to release of entrapped nutrients (Dekker and Richards, 1976), reduction of digesta viscosity (Machado et al., 2020), and improving nutrient digestibility including energy (Kiarie et al., 2014; Petry et al., 2020). In a study reported by Machado et al. (2020), digesta viscosity was reduced thus energy digestibility was improved (Chang et al., 2019; Nusairat et al., 2022). Furthermore, Machado et al. (2020) also reported that the use of probiotics inferred with enzymatic complex bacteria that can produce enzymes to hydrolyze NSP and improve intestinal environment. Therefore, in this study, the combination of xylanase with the probiotic may have combinedly lead to the improvement in the AME and AMEn in a way that the xylanase support was by providing the probiotics with the nutrients they need; not only by providing prebiotics but with providing energy as well. However, that does not replace or negate the inclusion of regularly used xylanases in the feed which are supplemented to release energy for the support of the host and are assigned an energy-contribution value thus help in reducing the cost of feed.

Supplementation of either DFM or monensin had a beneficial effect on intestinal lesion scores at 21 and 42 d, this confirms previous results by Nusairat et al. (2018; 2022) and Nusairat and Wang (2020), who reported the beneficial effect of xylanase and probiotic combination on reducing lesions caused by coccidia, while Zhou et al. (2016) reported that supplementing *B. licheniformis* to broilers challenged with *C. perfringens* improved FCR, reduced necrotic enteritis-associated oxidative stress and liver damage. Furthermore, *B. amyloliquefaciens* have been shown to reduce lesion scores, intestinal *C. perfringens* counts, footpad dermatitis, improve FCR, flock uniformity, and carcass yield in birds challenged with *C. perfringens* and *Eimeria* species (Tsukahara et al., 2018; de Oliveira et al., 2019). These beneficial effects are achieved through the competitive exclusion as well as the production of antimicrobial compounds exerted by the Bacillus-based probiotics (Ogbuewu et al., 2022). The probiotic potential was further enhanced by its combination with xylanase (Nusairat and Wang, 2020) leading to the reduction in intestinal lesions. Monensin and other anticoccidial medications have been shown to reduce coccidial lesions (Hooge et al., 1999a; 1999b, 2000; Nooreh et al., 2021) as well.

Intestinal digesta load of *E. coli*, aerobic plate count (APC), and *C. perfringens*, cecal load of *E. tenella*, Salmonella incidence in intestinal digesta, and overall intestinal lesion score measured at 21 and 42 d of age were affected by the supplementation of either DFM or monensin, or their combination. Literature is limited around the interaction between probiotics and monensin, most of the work is focused on using feed additives of either herbs or probiotics as an alternative to antibiotic growth promotors. The combination of DFM and monensin lead to a numerical reduction beyond each product alone, indicating that the products did not negatively affect each other, rather cooperated to improve the gut health of the birds leading to decreased microbial load. Trela et al. (2020) reported that the interaction of Salinomycin, another ionophore, and *B. licheniformis* reduced *C. perfringens* in the ceca when tested in 36 d old broilers. Furthermore, in another study conducted by Kan et al. (2021) comparing *B. licheniformis* to enramycin (antibiotic) under a subclinical necrotic enteritis disease model, they observed that the probiotic was superior to the antibiotic in reducing intestinal lesion scores and cecal *C. perfringens* counts, while Knap et al. (2010) found *B. licheniformis* comparable to virginiamycin when compared under necrotic enteritis disease challenge in broilers.

Litter moisture increased as birds aged, which was expected due to excreta and moisture accumulation as birds got older, as well as house management including drinkers’ maintenance needs (Coufal et al., 2006). Reducing litter moisture can help in reducing incidence of footpad dermatitis (FPD) thus avoiding potential welfare violation; Wang et al. (1998) and Alabi et al. (2024) demonstrated that there was a positive correlation between litter moisture content, litter pH, and the incidence and severity of FPD among broiler birds. Supplementation of DFM had no effect on litter moisture, while monensin reduced litter moisture at 42 d by approximately 1 %, which could be due to a lower water intake in those birds as demonstrated in previous research (Ouart et al., 1995), compared to the control and DFM-supplemented birds.

In the current study, the reduction of *C. perfringens* in the litter, the leading cause of necrotic enteritis, was achieved by monensin supplementation at both 21 and 42 d, which could partially be related to lower litter moisture in those pens, while DFM supplementation tended to decrease *C. perfringens* counts at 21 d. Monensin has been reported to reduced *C. perfringens* (Elwinger et al., 1998; Vissiennon et al., 2000; Silva et al., 2009) in broilers. However, the concern of developing antimicrobial resistance and subsequently affecting human health has been raised under the One Health initiative, with combating antimicrobial resistance as a priority (Carresi et al., 2024) thus calling for rigorous analytical monitoring on antimicrobials to control bacterial cross-resistance. Such concerns have promoted for using alternative solutions that do not pose high risk under the One Health approach, such as the probiotics (Kulkarni et al., 2022).

Dietary supplementation of DFM or monensin either alone or in combinations was able to alleviate the negative effects from the challenges with *Eimeria* species and *C. perfringens* when spiked in the litter. DFM was also able to improve AME beyond monensin due to its xylanase component creating a synergistic effect of the xylanase and direct-fed microbials (DFM) combination, and the prebiotics produced by the action of such products, resulting in a novel combination capable of improving performance, reducing intestinal lesions and damage, thus promoting for healthy gut microflora that could aid in minimizing favorable conditions for *C. perfringens* to express its virulency. Therefore, DFM can be used as an alternative or in combination with monensin for improving performance and reducing pathogens load. Further research can elaborate on investigating multiple natural alternatives to antibiotics that could be used as a combination or sequential solution through the different stages of poultry production and minimize the reliance on coccidiostats thus the development of antibiotic resistance.

## CRediT authorship contribution statement

**Basheer Nusairat:** Writing – review & editing, Writing – original draft, Visualization, Validation, Supervision, Methodology, Investigation, Formal analysis, Data curation, Conceptualization. **Rasha Qudsieh:** Writing – review & editing, Project administration, Formal analysis. **Jeng-Jie Wang:** Writing – review & editing, Resources.

## Disclosures

The authors declare that they have no known competing financial interests or personal relationships that could have appeared to influence the work reported in this paper.
